# 1-Acetyl-4-phenyl-5a,6,7,8,9,9a-hexa­hydro-5*H*-1,5-benzodiazepin-2(1*H*)-one

**DOI:** 10.1107/S1600536809039932

**Published:** 2009-10-07

**Authors:** Hanane Benzeid, Nathalie Saffon, Bernard Garrigues, El Mokhtar Essassi, Seik Weng Ng

**Affiliations:** a Laboratoire de Chimie Organique Hétérocyclique, Pôle de Compétences Pharmacochimie, Université Mohammed V-Agdal, BP 1014 Avenue Ibn Batout, Rabat, Morocco; b Service Commun Rayons X, Université Paul Sabatier, Bâtiment 2R1, 118 route de Narbonne, 31062 Toulouse, France; c Hétérochimie Fondamentale et Appliquée, Université Paul Sabatier, UMR 5069, 118 Route de Narbonne, 31062 Toulouse, France; d Department of Chemistry, University of Malaya, 50603 Kuala Lumpur, Malaysia

## Abstract

The seven-membered ring of the title compound, C_17_H_20_N_2_O_2_, adopts an approximate boat conformation while the cyclo­hexyl ring adopts a chair conformation. In the crystal, adjacent mol­ecules are linked by N—H⋯O hydrogen bonds into a zigzag chain running along the *c* axis of the monoclinic unit cell.

## Related literature

For the crystal structures of anhydrous and hydrated 7-phenyl-1,2, 3,4-tetra­hydro-1,4-diazepin-5-ones, see: Clark *et al.* (1999 ▶); Chammache *et al.* (2001 ▶).
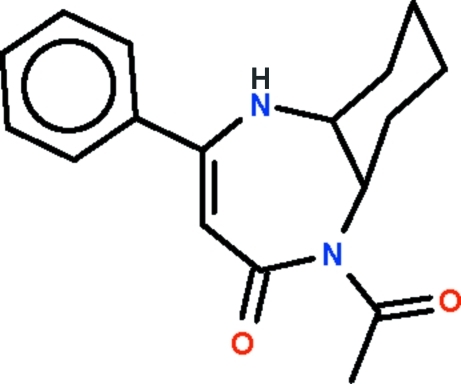


## Experimental

### 

#### Crystal data

C_17_H_20_N_2_O_2_
*M* *_r_* = 284.35
Monoclinic, 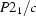

*a* = 9.6794 (2) Å
*b* = 14.0095 (3) Å
*c* = 11.2832 (2) Å
β = 98.053 (1)°
*V* = 1514.95 (5) Å^3^
*Z* = 4
Mo *K*α radiation
μ = 0.08 mm^−1^
*T* = 193 K
0.6 × 0.6 × 0.6 mm

#### Data collection

Bruker APEXII diffractometer
Absorption correction: none
27399 measured reflections
4591 independent reflections
3878 reflections with *I* > 2σ(*I*)
*R*_int_ = 0.024

#### Refinement

*R*[*F* ^2^ > 2σ(*F* ^2^)] = 0.046
*wR*(*F* ^2^) = 0.139
*S* = 1.05
4591 reflections
195 parameters
1 restraint
H atoms treated by a mixture of independent and constrained refinement
Δρ_max_ = 0.38 e Å^−3^
Δρ_min_ = −0.30 e Å^−3^

### 

Data collection: *APEX2* (Bruker, 2005 ▶); cell refinement: *SAINT* (Bruker, 2005 ▶); data reduction: *SAINT*; program(s) used to solve structure: *SHELXS97* (Sheldrick, 2008 ▶); program(s) used to refine structure: *SHELXL97* (Sheldrick, 2008 ▶); molecular graphics: *X-SEED* (Barbour, 2001 ▶); software used to prepare material for publication: *publCIF* (Westrip, 2009 ▶).

## Supplementary Material

Crystal structure: contains datablocks global, I. DOI: 10.1107/S1600536809039932/tk2546sup1.cif

Structure factors: contains datablocks I. DOI: 10.1107/S1600536809039932/tk2546Isup2.hkl

Additional supplementary materials:  crystallographic information; 3D view; checkCIF report

## Figures and Tables

**Table 1:** Hydrogen-bond geometry (Å, °)

*D*—H⋯*A*	*D*—H	H⋯*A*	*D*⋯*A*	*D*—H⋯*A*
N2—H2⋯O1^i^	0.88 (1)	2.001 (9)	2.854 (1)	164 (1)

Symmetry code: (i) 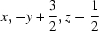.

**Fig. 1.**
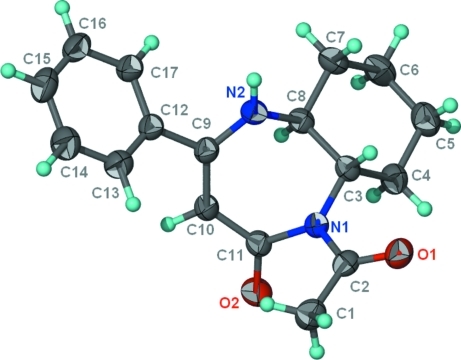
Thermal ellipsoid plot (Barbour, 2001) of C17H20N2O2 at the 70% probability level; hydrogen atoms are drawn as spheres of arbitrary radius.

## supplementary crystallographic information

### Experimental

4-Phenyl-5*a*,6,7,8,9,9*a*-hexahydro-1*H*-benzo[*b*][1,4]diazepin-2(3*H*)-one (1 g) was refluxed in acetic acid (20 ml) for 12 h. The precipate was collected and recrystallized from ethanol to afford colorless crystals in 90% yield.

### Refinement

Carbon-bound H-atoms were placed in calculated positions (C—H 0.95 to 0.99 Å) and were included in the refinement in the riding model approximation, with *U*_iso_(H) set to 1.2–1.5*U*_eq_(C). The amino H-atom was located in a difference Fourier map and was refined with an N–H 0.88±0.01 Å restraint.

### Crystal data

**Table:**

C_17_H_20_N_2_O_2_	*F*(000) = 608
*M**_r_* = 284.35	*D*_x_ = 1.247 Mg m^−^^3^
Monoclinic, *P*2_1_/*c*	Mo *K*α radiation, λ = 0.71073 Å
Hall symbol: -P 2ybc	Cell parameters from 9928 reflections
*a* = 9.6794 (2) Å	θ = 5.2–30.6°
*b* = 14.0095 (3) Å	µ = 0.08 mm^−^^1^
*c* = 11.2832 (2) Å	*T* = 193 K
β = 98.053 (1)°	Block, colorless
*V* = 1514.95 (5) Å^3^	0.6 × 0.6 × 0.6 mm
*Z* = 4	

### Data collection

**Table:**

Bruker APEXII diffractometer	3878 reflections with *I* > 2σ(*I*)
Radiation source: fine-focus sealed tube	*R*_int_ = 0.024
graphite	θ_max_ = 30.5°, θ_min_ = 5.2°
φ and ω scans	*h* = −13→13
27399 measured reflections	*k* = 0→20
4591 independent reflections	*l* = 0→16

### Refinement

**Table:**

Refinement on *F*^2^	Primary atom site location: structure-invariant direct methods
Least-squares matrix: full	Secondary atom site location: difference Fourier map
*R*[*F*^2^ > 2σ(*F*^2^)] = 0.046	Hydrogen site location: inferred from neighbouring sites
*wR*(*F*^2^) = 0.139	H atoms treated by a mixture of independent and constrained refinement
*S* = 1.05	*w* = 1/[σ^2^(*F*_o_^2^) + (0.0824*P*)^2^ + 0.2971*P*] where *P* = (*F*_o_^2^ + 2*F*_c_^2^)/3
4591 reflections	(Δ/σ)_max_ = 0.001
195 parameters	Δρ_max_ = 0.38 e Å^−^^3^
1 restraint	Δρ_min_ = −0.30 e Å^−^^3^

### Fractional atomic coordinates and isotropic or equivalent isotropic displacement parameters (Å^2^)

**Table:**

	*x*	*y*	*z*	*U*_iso_*/*U*_eq_	
O1	0.38581 (9)	0.66240 (6)	0.46797 (7)	0.03445 (19)	
O2	0.32929 (9)	0.42092 (6)	0.27399 (8)	0.0390 (2)	
N1	0.40005 (8)	0.57744 (6)	0.29988 (7)	0.02455 (17)	
N2	0.44012 (9)	0.65352 (6)	0.07259 (8)	0.02644 (18)	
H2	0.4411 (15)	0.7098 (7)	0.0384 (12)	0.038 (4)*	
C1	0.18397 (12)	0.57212 (10)	0.39317 (10)	0.0398 (3)	
H1A	0.1299	0.6227	0.4252	0.060*	
H1B	0.1843	0.5152	0.4436	0.060*	
H1C	0.1417	0.5566	0.3114	0.060*	
C2	0.33153 (10)	0.60571 (7)	0.39210 (8)	0.0266 (2)	
C3	0.53214 (10)	0.62624 (7)	0.28648 (9)	0.02480 (19)	
H3	0.5220	0.6951	0.3071	0.030*	
C4	0.65550 (11)	0.58553 (8)	0.37093 (10)	0.0321 (2)	
H4A	0.6663	0.5168	0.3538	0.039*	
H4B	0.6375	0.5918	0.4548	0.039*	
C5	0.78980 (12)	0.63873 (10)	0.35493 (12)	0.0412 (3)	
H5A	0.8697	0.6087	0.4059	0.049*	
H5B	0.7828	0.7058	0.3812	0.049*	
C6	0.81573 (12)	0.63687 (9)	0.22446 (12)	0.0382 (3)	
H6A	0.8330	0.5703	0.2009	0.046*	
H6B	0.8999	0.6750	0.2160	0.046*	
C7	0.69074 (11)	0.67727 (8)	0.14134 (10)	0.0322 (2)	
H7A	0.7086	0.6732	0.0572	0.039*	
H7B	0.6774	0.7453	0.1607	0.039*	
C8	0.55891 (10)	0.62073 (7)	0.15649 (9)	0.02496 (19)	
H8	0.5762	0.5523	0.1379	0.030*	
C9	0.33011 (10)	0.59655 (7)	0.03819 (8)	0.02568 (19)	
C10	0.30297 (11)	0.51532 (7)	0.10041 (9)	0.0280 (2)	
H10	0.2498	0.4668	0.0563	0.034*	
C11	0.34703 (10)	0.49695 (7)	0.22489 (9)	0.02630 (19)	
C12	0.24115 (11)	0.62065 (7)	−0.07600 (9)	0.0269 (2)	
C13	0.09583 (12)	0.61485 (8)	−0.08454 (10)	0.0333 (2)	
H13	0.0541	0.5972	−0.0164	0.040*	
C14	0.01201 (13)	0.63481 (9)	−0.19226 (11)	0.0390 (3)	
H14	−0.0867	0.6311	−0.1976	0.047*	
C15	0.07343 (14)	0.66027 (9)	−0.29194 (10)	0.0400 (3)	
H15	0.0164	0.6734	−0.3657	0.048*	
C16	0.21775 (14)	0.66671 (8)	−0.28440 (10)	0.0365 (2)	
H16	0.2590	0.6844	−0.3527	0.044*	
C17	0.30146 (12)	0.64719 (7)	−0.17679 (9)	0.0308 (2)	
H17	0.4000	0.6519	−0.1716	0.037*	

### Atomic displacement parameters (Å^2^)

**Table:**

	*U*^11^	*U*^22^	*U*^33^	*U*^12^	*U*^13^	*U*^23^
O1	0.0456 (5)	0.0305 (4)	0.0279 (4)	−0.0012 (3)	0.0075 (3)	−0.0051 (3)
O2	0.0502 (5)	0.0266 (4)	0.0384 (4)	−0.0081 (3)	−0.0005 (4)	0.0075 (3)
N1	0.0264 (4)	0.0238 (4)	0.0234 (4)	−0.0018 (3)	0.0033 (3)	−0.0015 (3)
N2	0.0308 (4)	0.0209 (4)	0.0275 (4)	−0.0017 (3)	0.0038 (3)	0.0017 (3)
C1	0.0286 (5)	0.0589 (7)	0.0326 (5)	−0.0009 (5)	0.0063 (4)	0.0009 (5)
C2	0.0301 (5)	0.0263 (4)	0.0236 (4)	0.0030 (3)	0.0038 (3)	0.0026 (3)
C3	0.0256 (4)	0.0214 (4)	0.0273 (4)	−0.0012 (3)	0.0034 (3)	−0.0019 (3)
C4	0.0281 (5)	0.0344 (5)	0.0324 (5)	0.0006 (4)	−0.0010 (4)	−0.0002 (4)
C5	0.0286 (5)	0.0494 (7)	0.0440 (6)	−0.0053 (5)	−0.0006 (4)	−0.0051 (5)
C6	0.0272 (5)	0.0390 (6)	0.0492 (7)	−0.0038 (4)	0.0080 (4)	−0.0051 (5)
C7	0.0314 (5)	0.0278 (5)	0.0387 (5)	−0.0050 (4)	0.0096 (4)	−0.0028 (4)
C8	0.0258 (4)	0.0208 (4)	0.0286 (4)	−0.0003 (3)	0.0049 (3)	−0.0016 (3)
C9	0.0310 (5)	0.0233 (4)	0.0229 (4)	−0.0006 (3)	0.0047 (3)	−0.0026 (3)
C10	0.0357 (5)	0.0228 (4)	0.0251 (4)	−0.0053 (3)	0.0030 (4)	−0.0028 (3)
C11	0.0290 (4)	0.0219 (4)	0.0277 (4)	−0.0014 (3)	0.0029 (3)	−0.0005 (3)
C12	0.0326 (5)	0.0246 (4)	0.0235 (4)	−0.0022 (3)	0.0036 (3)	−0.0012 (3)
C13	0.0335 (5)	0.0366 (5)	0.0300 (5)	−0.0015 (4)	0.0056 (4)	0.0012 (4)
C14	0.0347 (5)	0.0422 (6)	0.0383 (6)	0.0006 (4)	−0.0006 (4)	0.0002 (5)
C15	0.0506 (7)	0.0390 (6)	0.0277 (5)	0.0019 (5)	−0.0041 (5)	−0.0006 (4)
C16	0.0519 (7)	0.0348 (5)	0.0233 (4)	−0.0036 (5)	0.0067 (4)	−0.0006 (4)
C17	0.0383 (5)	0.0283 (5)	0.0263 (4)	−0.0042 (4)	0.0065 (4)	−0.0023 (4)

### Geometric parameters (Å, °)

**Table:**

O1—C2	1.2303 (13)	C6—H6A	0.9900
O2—C11	1.2238 (12)	C6—H6B	0.9900
N1—C2	1.3686 (12)	C7—C8	1.5317 (14)
N1—C11	1.4591 (12)	C7—H7A	0.9900
N1—C3	1.4761 (12)	C7—H7B	0.9900
N2—C9	1.3438 (13)	C8—H8	1.0000
N2—C8	1.4574 (13)	C9—C10	1.3815 (13)
N2—H2	0.878 (9)	C9—C12	1.4849 (14)
C1—C2	1.5055 (15)	C10—C11	1.4328 (14)
C1—H1A	0.9800	C10—H10	0.9500
C1—H1B	0.9800	C12—C13	1.3987 (15)
C1—H1C	0.9800	C12—C17	1.3995 (14)
C3—C4	1.5298 (14)	C13—C14	1.3919 (16)
C3—C8	1.5268 (13)	C13—H13	0.9500
C3—H3	1.0000	C14—C15	1.3904 (17)
C4—C5	1.5309 (16)	C14—H14	0.9500
C4—H4A	0.9900	C15—C16	1.3904 (19)
C4—H4B	0.9900	C15—H15	0.9500
C5—C6	1.5279 (18)	C16—C17	1.3892 (16)
C5—H5A	0.9900	C16—H16	0.9500
C5—H5B	0.9900	C17—H17	0.9500
C6—C7	1.5315 (17)		
			
C2—N1—C11	119.73 (8)	C6—C7—C8	109.85 (9)
C2—N1—C3	117.57 (8)	C6—C7—H7A	109.7
C11—N1—C3	122.54 (8)	C8—C7—H7A	109.7
C9—N2—C8	121.44 (8)	C6—C7—H7B	109.7
C9—N2—H2	117.6 (10)	C8—C7—H7B	109.7
C8—N2—H2	120.8 (10)	H7A—C7—H7B	108.2
C2—C1—H1A	109.5	N2—C8—C3	112.54 (8)
C2—C1—H1B	109.5	N2—C8—C7	111.00 (8)
H1A—C1—H1B	109.5	C3—C8—C7	109.71 (8)
C2—C1—H1C	109.5	N2—C8—H8	107.8
H1A—C1—H1C	109.5	C3—C8—H8	107.8
H1B—C1—H1C	109.5	C7—C8—H8	107.8
O1—C2—N1	120.71 (9)	N2—C9—C10	122.81 (9)
O1—C2—C1	120.62 (9)	N2—C9—C12	117.08 (8)
N1—C2—C1	118.49 (9)	C10—C9—C12	119.99 (9)
N1—C3—C4	112.03 (8)	C9—C10—C11	126.47 (9)
N1—C3—C8	109.94 (7)	C9—C10—H10	116.8
C4—C3—C8	110.90 (8)	C11—C10—H10	116.8
N1—C3—H3	107.9	O2—C11—C10	123.97 (9)
C4—C3—H3	107.9	O2—C11—N1	118.06 (9)
C8—C3—H3	107.9	C10—C11—N1	117.51 (8)
C3—C4—C5	110.28 (9)	C13—C12—C17	119.30 (10)
C3—C4—H4A	109.6	C13—C12—C9	120.13 (9)
C5—C4—H4A	109.6	C17—C12—C9	120.55 (9)
C3—C4—H4B	109.6	C14—C13—C12	120.40 (10)
C5—C4—H4B	109.6	C14—C13—H13	119.8
H4A—C4—H4B	108.1	C12—C13—H13	119.8
C4—C5—C6	111.23 (10)	C15—C14—C13	119.66 (11)
C4—C5—H5A	109.4	C15—C14—H14	120.2
C6—C5—H5A	109.4	C13—C14—H14	120.2
C4—C5—H5B	109.4	C14—C15—C16	120.46 (11)
C6—C5—H5B	109.4	C14—C15—H15	119.8
H5A—C5—H5B	108.0	C16—C15—H15	119.8
C5—C6—C7	111.19 (9)	C17—C16—C15	119.92 (10)
C5—C6—H6A	109.4	C17—C16—H16	120.0
C7—C6—H6A	109.4	C15—C16—H16	120.0
C5—C6—H6B	109.4	C16—C17—C12	120.26 (11)
C7—C6—H6B	109.4	C16—C17—H17	119.9
H6A—C6—H6B	108.0	C12—C17—H17	119.9
			
C11—N1—C2—O1	168.01 (9)	C8—N2—C9—C12	−158.47 (9)
C3—N1—C2—O1	−7.51 (14)	N2—C9—C10—C11	24.81 (16)
C11—N1—C2—C1	−16.81 (14)	C12—C9—C10—C11	−159.20 (10)
C3—N1—C2—C1	167.66 (9)	C9—C10—C11—O2	−174.30 (11)
C2—N1—C3—C4	81.59 (10)	C9—C10—C11—N1	13.72 (15)
C11—N1—C3—C4	−93.80 (10)	C2—N1—C11—O2	−55.02 (13)
C2—N1—C3—C8	−154.61 (8)	C3—N1—C11—O2	120.27 (11)
C11—N1—C3—C8	30.00 (11)	C2—N1—C11—C10	117.44 (10)
N1—C3—C4—C5	−179.46 (9)	C3—N1—C11—C10	−67.27 (12)
C8—C3—C4—C5	57.29 (11)	N2—C9—C12—C13	−138.24 (10)
C3—C4—C5—C6	−55.11 (13)	C10—C9—C12—C13	45.54 (14)
C4—C5—C6—C7	55.70 (14)	N2—C9—C12—C17	43.26 (13)
C5—C6—C7—C8	−57.34 (12)	C10—C9—C12—C17	−132.96 (10)
C9—N2—C8—C3	−80.75 (11)	C17—C12—C13—C14	0.29 (16)
C9—N2—C8—C7	155.86 (9)	C9—C12—C13—C14	−178.23 (10)
N1—C3—C8—N2	52.05 (10)	C12—C13—C14—C15	0.27 (18)
C4—C3—C8—N2	176.50 (8)	C13—C14—C15—C16	−0.56 (19)
N1—C3—C8—C7	176.16 (8)	C14—C15—C16—C17	0.29 (18)
C4—C3—C8—C7	−59.39 (10)	C15—C16—C17—C12	0.27 (17)
C6—C7—C8—N2	−176.16 (8)	C13—C12—C17—C16	−0.56 (16)
C6—C7—C8—C3	58.84 (11)	C9—C12—C17—C16	177.95 (9)
C8—N2—C9—C10	17.64 (14)		

### Hydrogen-bond geometry (Å, °)

**Table:**

*D*—H···*A*	*D*—H	H···*A*	*D*···*A*	*D*—H···*A*
N2—H2···O1^i^	0.88 (1)	2.00 (1)	2.854 (1)	164 (1)

Symmetry codes: (i) *x*, −*y*+3/2, *z*−1/2.
